# Targeting regulatory T cell plasticity in gastric cancer: a unified bimodal model and precision immunotherapy paradigm: a hypothesis-generating review

**DOI:** 10.3389/fonc.2026.1882680

**Published:** 2026-07-22

**Authors:** PengFei Zhou, DanYa Wang, HaiYa Zhang, FengLin Zhang, ZhiTong Li, HongQing Xi

**Affiliations:** 1 Medical School of Chinese People's Liberation Army (PLA), Beijing, China; 2 Department of General Surgery, The First Medical Center of Chinese People's Liberation Army (PLA) General Hospital, Beijing, China

**Keywords:** fragile-like Treg, gastric cancer, immunotherapy, Treg cell, tumor microenvironment

## Abstract

In contrast to other solid tumors, gastric cancer exhibits a particularly high dependence on regulatory T cell (Treg)-mediated immunosuppression. This reliance represents a critical driver of both primary and acquired resistance to programmed death-1/ligand-1(PD-1/PD-L1) inhibitors. By integrating existing single-cell, spatial transcriptomic, and clinical evidence, this review proposes a unified bimodal model of the Treg tumor microenvironment. Within this framework, the immune status in gastric cancer is determined by the dynamic equilibrium between immunosuppressive stable-type Tregs and pro-inflammatory fragile-type Tregs. H. pylori is posited as the master upstream regulator of this balance, capable of driving Treg differentiation toward either stability or fragility through temporally specific signaling pathways. Based on this framework, we introduce the Treg Stability-Fragility(Treg-SF) score, which incorporates Treg functional state, H. pylori infection status, and CD8^+^T cell density as independently weighted variables within a unified model to predict immunotherapy efficacy. Furthermore, this review outlines three mechanism-based combination therapeutic strategies that target Treg plasticity, along with a detailed preclinical validation roadmap and risk–benefit analysis. These proposals provide an actionable translational roadmap to overcome Treg-mediated immune resistance and improve clinical outcomes in gastric cancer.

## 1 Introduction

Although PD-1/PD-L1 inhibitors have improved the prognosis of a subset of patients with advanced gastric cancer, their clinical benefit remains limited. In unselected populations, monotherapy yields an objective response rate (ORR) of only 12–18%. Even in patients with a PD-L1 combined positive score (CPS) ≥10, the ORR is limited to 20–25%. More than 70% of patients demonstrate primary resistance, and the majority of initial responders eventually acquire secondary resistance (1, 2). Regulatory T cells (Tregs) are well-established as a potent immunosuppressive T cell subset that inhibits CD8^+^ T cell activity and contributes substantially to tumor immune evasion (2, 3). Tumor-infiltrating Tregs serve as a pivotal component of the immunosuppressive network, establishing both physical and functional barriers that impede effector T cell infiltration and activation (2, 3).

Early targeted therapies targeting regulatory T cells (Tregs), such as depleting CD25^+^ cells via anti-CD25 monoclonal antibodies, have not been associated with severe autoimmune toxicity in clinical trials. However, their clinical development in the treatment of solid tumors has been severely hampered by the challenge of precisely distinguishing between tumor-associated pathogenic Tregs and peripheral homeostatic Tregs (4). Single-cell RNA sequencing (scRNA-seq) and spatial transcriptomics have completely revolutionized our understanding of regulatory T cell (Treg) biology, revealing that Tregs constitute a highly heterogeneous and plastic cell population rather than a functionally uniform cell lineage (5, 6). Notably, the discovery of fragile Tregs has challenged the conventional wisdom (7): this subset exhibits reduced expression of forkhead box protein P3 (Foxp3),co-expresses T-bet and interferon-γ (IFN-γ), displays impaired immunosuppressive function, and exhibits potential pro-inflammatory activity, offering a novel perspective for precision cancer immunotherapy.

This review is a comprehensive hypothesis-generating study that integrates available clinical, translational, and multi-omics data to propose a novel conceptual framework for understanding Treg heterogeneity in gastric cancer. It first summarizes the current consensus regarding Treg subsets in gastric cancer, highlights landmark single-cell datasets, and compares Treg heterogeneity with that in other solid tumors. It then elaborates on the bimodal Treg microenvironment model and the potential regulatory role of H. pylori infection, clearly defining the stable/fragile Treg (S/F) ratio and noting that this ratio remains to be experimentally validated. It proposes the Treg-SF score as a conceptual biomarker and systematically evaluates current Treg-targeted therapeutic strategies. Finally, it presents three verifiable combination therapeutic regimens with detailed preclinical validation plans, and critically discusses the limitations of existing evidence and future research priorities. Furthermore, Treg instability is not unique to tumor immunity but also contributes to the development of autoimmune diseases (8). The perspective presented herein can serve as an illustrative example of the “mirror regulation” principle in both oncology and autoimmunity. Such an interdisciplinary, unifying view may open new avenues for bidirectional translation in precision immunotherapy.

## 2 Treg heterogeneity in Gastric Cancer

By constructing a pan-cancer single-cell transcriptomic atlas of tumor-infiltrating T cells, Zheng et al. (6) demonstrated the presence of functionally distinct Treg subsets in the gastric cancer tumor microenvironment (TME). This landmark study, published in Science, analyzed approximately 397,000 T cells across 21 cancer types, identifying one dominant immunosuppressive Treg cluster and a second, smaller, transcriptionally unique anti-tumor Treg cluster.

Subsequent scRNA-seq studies have replicated and validated these findings, confirming that gastric cancer exhibits greater intratumoral Treg enrichment and a higher proportion of Tregs among CD4^+^ T cells than other solid tumors (9, 10).

### 2.1 Stable suppressive Tregs: the core driver of tumor immune evasion

1.CCR8^+^ Tregs: This is the most highly enriched Treg subset in tumors, preferentially localized at the invasive front.CCR8^+^ Tregs are highly enriched among tumor-infiltrating Tregs in gastric cancer (reported at 40%–60%), and their density is negatively correlated with CD8^+^ T-cell infiltration and patient survival. This subset is recruited by CCL1 secreted by tumor cells and cancer-associated fibroblasts (CAFs), making it one of the most promising targets for next-generation Treg-targeted therapy (11).

2.LAG3^+^ Tregs: This subset is induced by tumor-derived serglycin via the CD44/TGFβRI/Smad3 pathway and mediates PD-1-independent immunosuppression. It is closely associated with the immunosuppressive microenvironment and tumor progression in gastric cancer (12).

3. TNF^+^ Tregs: This functionally distinct subset directly promotes tumor progression by secreting IL-13. IL-13 activates the STAT3 signaling pathway in gastric cancer cells and upregulates stemness genes, thereby fueling tumor growth (13).

All three Treg subsets mentioned above are characterized by high Foxp3 expression, full demethylation of the Treg-specific demethylated region (TSDR), and potent immunosuppressive activity, consistent with the biological features of Tregs in other solid tumors (3, 14).

### 2.2 Fragile-like Tregs

Fragile-like Tregs represent a unique cell subset that retains partial transcriptional signatures of suppressive Tregs while exerting pro-inflammatory effects (15). The first pan-cancer single-cell atlas of tumor-infiltrating T cells, published by Zheng et al., has provided a pivotal framework for understanding Treg heterogeneity in gastric cancer (6, 16). This subset is defined as having reduced but detectable Foxp3 expression, co-expressing the Th1 transcription factor T-bet and the effector cytokine IFN-γ, with partial loss of suppressive function (7).

The molecular mechanisms driving Treg fragility have been most thoroughly investigated in melanoma and breast cancer. The pioneering work by Overacre-Delgoffe et al. confirmed that IFN-γ triggers Treg instability in the tumor microenvironment and drives the formation of the fragile phenotype (7). At the signaling pathway level, Koch et al. (17) demonstrated that IFN-γ induces Foxp3^+^ Tregs to upregulate T-bet, endowing them with unique homeostatic and migratory properties in a Th1-inflammatory milieu. Gangaplara et al. further revealed that type I interferon signaling directly impairs the suppressive function of Tregs (18). Liaskou et al. elucidated the pivotal role of the IL-12-STAT4 pathway in driving Treg polarization toward a Th1-like phenotype (19). At the epigenetic level, epigenetic regulators such as TET proteins are essential for maintaining Treg stability, and strategies to induce fragility have provided new insights into cancer immunotherapy (8). Although we infer that these mechanisms are conserved in gastric cancer, direct functional validation in gastric cancer-specific models is still lacking (20). Furthermore, the true identity of fragile Tregs in the gastric cancer microenvironment remains unclear. Whether their pro-inflammatory effect represents a gain-of-function or a concomitant phenomenon of Treg identity loss still needs to be strictly distinguished by experiments such as lineage tracing.

Functional validation of fragile-like Tregs is predominantly based on melanoma and breast cancer models, while *in situ* functional evidence in gastric cancer remains insufficient. Two analytical strategies using public single-cell transcriptomic datasets can be adopted to address the controversy over whether these cells represent transient intermediates or stable subsets without conducting new experiments: RNA velocity kinetic analysis (21, 22): Using published gastric cancer single-cell RNA sequencing (scRNA-seq) datasets (e.g., GSE183904, GSE167297), we can determine the differentiation trajectory of Foxp3low T-bet+ cells according to the kinetic direction of spliced and unspliced transcripts. If the velocity vectors extend from canonical Foxp3high Tregs to this subset and no terminal branches leading to Th1 cells are observed, this would demonstrate that these cells are stable functional subsets rather than transient intermediates. TCR clonotype tracing analysis: Quantify the proportion of shared TCR clonotypes among suppressive Tregs, fragile-like Tregs, Th1 cells, and CD8+ T cells (3, 6). Fragile-like Tregs exhibit dominant clonal expansion independently and share fewer than 10% of TCR clonotypes with Th1 cells; they can be confirmed as a distinct cell lineage rather than Th1-like intermediates. Additionally, a negative correlation between the extent of clonal expansion and tumor stage can serve as indirect evidence of their immunosuppressive capacity. Supplementary spatial evidence: Multiplex immunohistochemistry (mIHC) analysis reveals that the expression of the proliferation marker MKI6 is markedly reduced in CD8^+^ T cells within regions where fragile-like Tregs and CD8^+^ T cells are spatially colocalized. This finding indirectly validates the immunosuppressive function of fragile-like Tregs. The analytical approach described above has been widely applied in studies on the spatial heterogeneity of tumor-associated macrophages (TAMs) in gastric cancer (23, 24).

Our preliminary reanalysis of the GSE183904 dataset — a large-scale single-cell atlas of gastric cancer encompassing over 200,000 cells from 31 patients across all clinical stages and both major histologic subtypes — provides initial descriptive support for the presence of fragile-like Tregs in the gastric tumor microenvironment (2). In this independent cohort, we identified a discrete subpopulation of CD4+ T cells marked by moderately reduced Foxp3 expression alongside low-level co-expression of TBX21 and IFNG, matching the core transcriptional signature of fragile-like Tregs. On UMAP embedding, this subset localizes to a continuous intermediate position between canonical Foxp3high immunosuppressive Tregs and conventional Th1 effector cells, and preliminary trajectory inference suggests a differentiation direction originating from mature suppressive Tregs rather than reverse differentiation from Th1 cells. Notably, the original study characterizing this dataset reported a well-defined IL2RA+STAT3+ Treg cluster (LT5), and our reannotation further resolves the internal functional heterogeneity within this Treg population. While these descriptive observations offer cross-cohort support for our proposed bimodal framework, we emphasize that they remain preliminary. Definitive confirmation of lineage stability and functional properties will require integrated TCR clonotype tracing and orthogonal *in vitro*/*in vivo* functional validation.

### 2.3 Cross-cancer comparison of Treg heterogeneity

While Treg infiltration heterogeneity is common across solid tumors, gastric cancer exhibits distinctive features, including extremely high intratumoral Treg infiltration. A pan-cancer immunogenomic analysis covering 33 cancer types revealed that gastric cancer is among the solid tumors with the highest intratumoral Treg enrichment, and its Treg infiltration level ranks among the top in gastrointestinal cancers (25–27). CCR8^+^ Tregs are dominant: Studies have reported that CCR8^+^ Tregs account for a remarkably high proportion of tumor-infiltrating Tregs in gastric cancer (11). This stands in sharp contrast to the relatively low expression level of this subset in other solid tumors, and this high abundance endows the gastric cancer tumor microenvironment with strong immunosuppressive properties (25). Unlike most other solid tumors, gastric cancer is uniquely regulated by the microbiota, with H. pylori infection profoundly affecting Tregs (28, 29). H. pylori infection can induce the production of immunosuppressive factors such as IL-10, and its own VacA protein targets myeloid cells in the gastric mucosa, disrupts the Th17/Treg balance induced by dendritic cells, reshapes Treg homeostasis in the tumor microenvironment, and exerts a bidirectional regulatory effect on Treg stability (28, 30).

**Figure 1:**
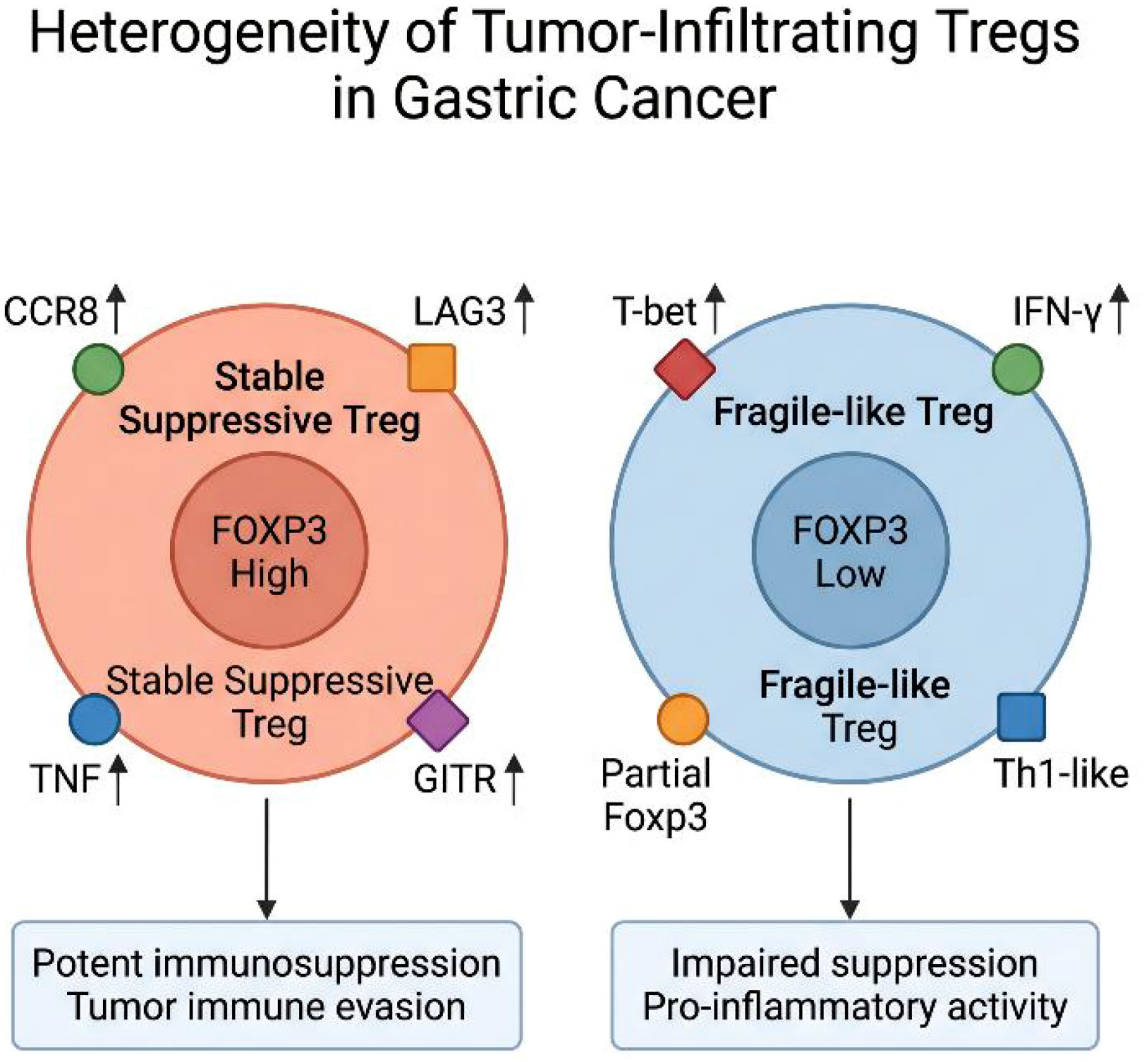
Heterogeneity of tumor−infiltrating regulatory T cells in gastric cancer.

In gastric cancer, tumor-infiltrating regulatory T cells (Tregs) do not constitute a homogeneous entity; rather, they encompass transcriptionally and functionally diverse subpopulations that can be broadly classified into stable suppressive Tregs and fragile-like Tregs (**Figure 1**). Stable suppressive Tregs are defined by robust expression of forkhead box protein P3 (Foxp3), complete demethylation of the Treg-specific demethylated region, and potent immunosuppressive capabilities. Within this group, CCR8^+^Tregs are the predominant subset, preferentially accumulating at the invasive margin, where they are recruited by CCL1 secreted by both tumor cells and cancer-associated fibroblasts. Notably, the abundance of these cells inversely correlates with CD8^+^T cell infiltration and patient prognosis. Meanwhile, LAG3^+^Tregs, induced through tumor-derived serglycin via the CD44/TGFβRI/Smad3 signaling axis, exert PD-1-independent immunosuppression. Distinctly, TNF^+^ Tregs promote tumor stemness by secreting IL-13, which activates STAT3 signaling within malignant cells. Conversely, fragile-like Tregs maintain residual Foxp3 levels but co-express the Th1 lineage transcription factor T-bet and the effector cytokine interferon-γ (IFN-γ). This phenotype, characterized as Foxp3lowT-bet^+^IFN-γ^+^, reflects impaired suppressive function and the acquisition of pro-inflammatory properties, likely resulting from interferon-mediated destabilization of the Treg lineage. The functional dichotomy between these two major categories provides a critical framework for elucidating Treg plasticity in the context of gastric malignancy.

### 2.4 Preclinical insights from animal models

Groundbreaking studies using melanoma and breast cancer models have elucidated the mechanisms underlying Treg fragility. For instance, IFN-γ can upregulate T-bet in Foxp3^+^ Tregs and impair their suppressive functions. Type I interferon signaling and the IL-12-STAT4 pathway are also involved in regulating the pro-inflammatory polarization of Tregs. These classic findings provide important mechanistic hypotheses for understanding Treg plasticity. In preclinical research, transgenic mouse models, including Foxp3 reporter mice and T-bet reporter mice, are widely used to track phenotypic shifts and fate decisions of Tregs across diverse tumor microenvironments. Relevant results have confirmed that Tregs are not terminally differentiated cells and can undergo functional transformation in response to local inflammatory cues.

Although these animal models have greatly advanced our understanding of Treg biology, gastric cancer-specific models remain scarce. Most existing animal studies on gastric cancer focus on validating individual signaling pathways within the tumor microenvironment. For example, mouse models of peritoneal metastasis have demonstrated that the hyaluronic acid-CD44 axis drives Treg differentiation via the CD44-IQGAP1-RAC1-SMAD pathway and facilitates the formation of an immunosuppressive microenvironment. Additionally, transgenic models of diffuse gastric cancer have indicated that Wnt signaling regulates the phenotypic transformation of signet ring cells into invasive phenotypes; nevertheless, the heterogeneity and plasticity of Tregs in these models have not been directly verified by lineage tracing and other functional experiments (31).

Collectively, the current hypothesis of Treg fragility in gastric cancer is mainly extrapolated from mechanistic discoveries in other cancer types. The lack of gastric cancer-specific transgenic animal models and *in vivo* functional validation represents a major limitation of the present study.

## 3 Innovative model: the bimodal Treg microenvironment model and the regulatory role of H. pylori

Building upon the aforementioned evidence, this review advances a unified bimodal model of the Treg microenvironment in gastric cancer. This framework contends that the immune landscape of gastric malignancies is governed not by the absolute number of regulatory T cells (Tregs), but rather by the dynamic equilibrium between stable, immunosuppressive Tregs and unstable, pro-inflammatory “fragile” subsets. We hypothesize that H. pylori infection serves as the pivotal modulator of this balance, orchestrating Treg stabilization or fragility through spatiotemporally distinct mechanisms. It is important to acknowledge that this conceptual model remains theoretical and necessitates validation via rigorous prospective clinical investigations.

### 3.1 Definition and quantification of the stable/fragile Treg ratio

The S/F ratio is defined as the ratio of the count of stable suppressive Tregs to the count of fragile Tregs in tumor tissue, and can be quantified using three complementary techniques:

#### 3.1.1 Single-cell RNA sequencing

The signature genes of immunosuppressive Tregs can be identified in the gene panel for effector Tregs, including Foxp3, CTLA4, CCR8, and TNFRSF1832 (32). Among them, CCR8 is selectively and highly expressed on immunosuppressive Tregs and serves as a key prognostic biomarker and immunotherapeutic target in gastric cancer (11, 23, 33). High expression of TNFRSF18 (GITR) is associated with potent immunosuppressive activity (34, 35).

The signature genes of fragile-like Tregs are characterized by low Foxp3 expression, accompanied by upregulated expression of TBX21 (encoding T-bet) and IFNG36. Deficiency of mitochondrial regulators such as CRIF1 can induce the generation of Foxp3low inflammatory non-suppressive Tregs, directly confirming the existence of this phenotype in tumors (36). Moreover, tumor antigen-specific Tregs downregulate Foxp3 and upregulate TBX21 and IFNG upon tumor infiltration, acquiring Th1-like features (36, 37).

Quantification method: The ratio of the number of cells expressing the immunosuppressive signature (Foxp3highCTLA4high CCR8^+^ TNFRSF18high TBX21^-^ IFNG^-^) to the number of cells expressing the fragile-like signature (Foxp3lowTBX21^+^ IFNG^+^) within the CD4^+^ T-cell cluster.

#### 3.1.2 Multiplex immunohistochemistry

Stable Tregs: Foxp3high T-bet^-^ CD4^+^ cells — distinguished from hybrid Tregs co-expressing T-bet by high Foxp3 expression (38).

Fragile Tregs: Foxp3low T-bet^+^ IFN-γ^+^ CD4^+^ cells — whose phenotype (low Foxp3 accompanied by IFN-γ secretion) has been independently validated in human tumor Treg studies (36).

Quantification method: Cell count per square millimeter of tumor tissue, averaged over 5–10 high-power fields. This standardized protocol has been successfully applied to gastric cancer clinical samples using mIHC, enabling combined quantification of CD8^+^ T cells and Foxp3^+^CD4^+^ T cells, and has been validated as a significant prognostic biomarker (39).

#### 3.1.3 Flow cytometry

Surface markers: CD4^+^, CD25^+^, CD127low (pan-Treg gating). This gating strategy has been validated by international consensus studies as the minimal essential marker panel for human Treg detection and is widely applied to peripheral blood mononuclear cells,tumor-draining lymph nodes, and fresh tumor tissues (40).

Intracellular marker definition:

Stable Tregs: Foxp3 high T-bet^-^, which fundamentally distinguishes conventional Foxp3^+^ Tregs from T-bet^+^ hybrid Tregs (40);

Fragile Tregs: Foxp3 lowT-bet^+^ IFN-γ^+^, a phenotype independently validated in human tumor Treg studies (34, 36).

Quantification method: The proportion of each subset within the CD4^+^ T cell population.

### 3.2 Dynamic equilibrium model

We hypothesize that the S/F ratio could serve as a key quantitative indicator for evaluating tumor immune status and immunotherapeutic response: an elevated S/F ratio corresponds to immune “cold” tumors, which are characterized by pervasive intratumoral immunosuppression and poor immunotherapeutic response in patients. This hypothesis is supported by clinical studies 23: a low CD8^+^/CCR8^+^ Treg ratio is significantly associated with unfavorable recurrence-free survival (RFS); pan-cancer data analysis also confirms that the CD8^+^/Treg ratio, as a prognostic biomarker, has superior predictive efficacy compared with the abundance index of a single immune subset (27). Conversely, a decreased S/F ratio indicates an immune “hot” tumor phenotype, in which the body can initiate a robust antitumor immune response, leading to a more favorable patient prognosis (15, 23, 27).

Notably, this dynamic balance can be dynamically regulated by therapeutic interventions. Clinical trial evidence indicates that PD-1 blockade can induce compensatory proliferation of CCR8^+^ stable Tregs, thereby enhancing the tumor immunosuppressive microenvironment, driving the development of acquired drug resistance, and potentially increasing the S/F ratio—which in turn validates the theory of Treg plasticity (15, 20, 41), Combination therapy with CCR8 antagonists and PD-1 inhibitors exerts a synergistic effect, which can inhibit the compensatory proliferation of immunosuppressive Tregs, reverse the immunosuppressive microenvironment, and reduce the S/F ratio (7, 41). In the field of chemotherapy, multiple studies have suggested that chemotherapeutic agents can reduce the total number of Tregs, reprogram their functional phenotypes (characterized by decreased Foxp3 expression and increased IFN-γ secretion), and thereby reduce the S/F ratio. Although specific clinical validation in gastric cancer is still lacking, pan-cancer research data have provided a solid conceptual basis for this regulatory pathway (42, 43).

### 3.3 H. pylori: a critical regulator of Treg bimodality

H. pylori infection is the most well-established risk factor for gastric cancer. Epidemiological studies have indicated that approximately 78% of non-cardia gastric cancers are attributable to H. pylori infection (44). Rather than exerting a unidirectional effect, H. pylori modulates Treg biology in a stage-dependent and opposing manner, enabling a dynamic switch between the induction of Treg fragility and the promotion of Treg stabilization.

1. Acute infection stage: H. pylori activates dendritic cells and macrophages via the TLR2/4 signaling pathway, inducing robust secretion of IL-12 and IFN-α. We therefore infer that this signaling cascade predominantly drives the induction of Treg fragility (29, 45, 46). Nevertheless, this initial pro-inflammatory response is usually insufficient to eliminate the pathogen, leading a subset of infected individuals to gradually develop chronic persistent infection (47).
2. Chronic infection stage: Persistent infection induces CagA-mediated gastric epithelial injury and activates the NF-κB and MAPK pathways, resulting in sustained secretion of TGF-β and IL-10 (47, 48). These cytokines promote the differentiation of immunosuppressive Tregs through Smad signaling and TET2/TET3-mediated TSDR demethylation (49, 50), ultimately leading to clonal expansion of suppressive Tregs and tumor immune escape (48, 51).

Strain differences further fine-tune this balance: studies have shown that CagA-positive H. pylori strains induce a higher level of stable Treg infiltration compared with CagA-negative strains, which is associated with an increased risk of gastric cancer (51). Our model hypothesizes that the dynamic changes in the S/F ratio over time determine the transition from anti-tumor immunity to immune escape. Thus, H. pylori, acting as a “bidirectional regulatory valve” for the S/F ratio, possesses dual capabilities: driving Treg fragility and enhancing immunosuppression. This is not a logical contradiction, but rather the inherent characteristic of H. pylori as the key master regulator.

**Figure 2:**
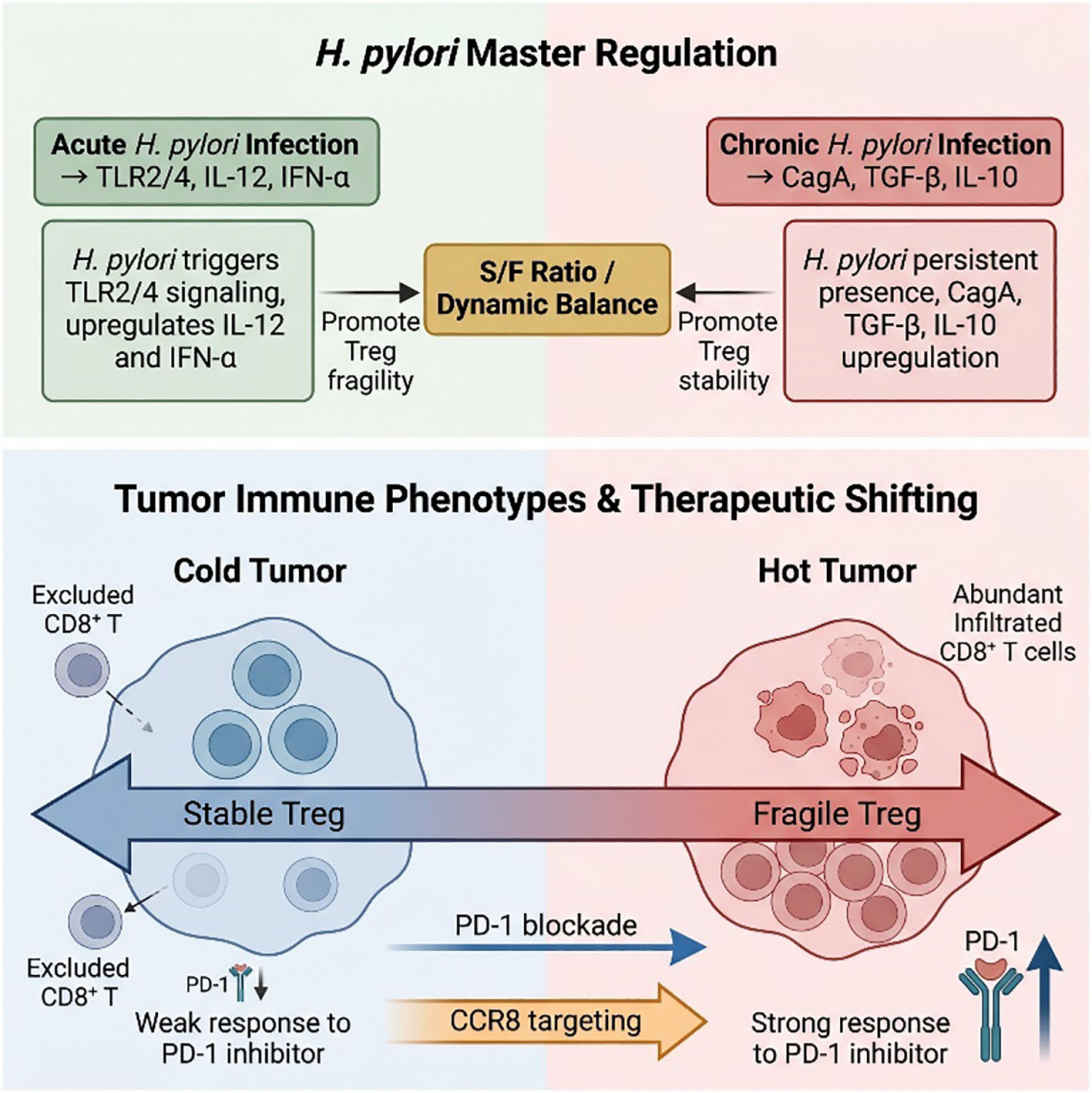
Bimodal Treg microenvironment model and the regulatory role of H. pylori.

The immunological landscape of gastric cancer is dictated not merely by the aggregate burden of regulatory T cells (Tregs), but rather by the dynamic interplay between immunosuppressive, stable Tregs and pro-inflammatory, fragile-like subsets (**Figure 2**). This balance is effectively captured by the stable-to-fragile Treg (S/F) ratio. Within this bimodal framework, a heightened S/F ratio signifies an immune-”cold” phenotype, marked by widespread immunosuppression, exclusion of CD8^+^T cells, and diminished responsiveness to PD-1 blockade. Conversely, a reduced S/F ratio indicates an immune-”hot” tumor environment, characterized by potent endogenous antitumor activity and superior outcomes following immunotherapy. H. pylori infection serves as a pivotal upstream modulator of this equilibrium, exerting stage-specific and contrasting influences. During acute infection, H. pylori stimulates dendritic cells and macrophages via TLR2/4-dependent mechanisms, triggering substantial secretion of IL-12 and IFN-α, which primarily promotes Treg fragility. In contrast, chronic persistent infection induces CagA-mediated gastric epithelial damage and sustained release of TGF-β and IL-10. These cytokines drive Treg stabilization through Smad signaling cascades and TET2/TET3-mediated epigenetic reprogramming, ultimately fostering the clonal expansion of suppressive Tregs and facilitating tumor immune evasion. Therapeutic strategies, including PD-1 inhibition or CCR8 targeting, can dynamically recalibrate this balance by selectively eliminating stable Tregs or inducing phenotypic fragility. Such interventions lower the S/F ratio, thereby shifting the tumor microenvironment toward an immune-activated state conducive to therapeutic efficacy.

#### 3.3.1 Controversy and balance discussion: timing and risks of H. pylori eradication

The association between H. pylori infection and the immunotherapeutic prognosis of gastric cancer remains highly controversial. Some studies have demonstrated that H. pylori-positive patients with advanced gastric cancer achieve better responses to PD-1 inhibitors and longer immune-related progression-free survival (52). However, other well-designed clinical investigations have yielded opposite results, showing significantly shorter overall survival (OS) in H. pylori-positive cases (53).

This apparent contradiction can be well explained by the Treg bimodal model. H. pylori exerts bidirectional regulation on the S/F ratio, with the ultimate outcome determined by infection stage, bacterial virulence, and host immune background. When infection drives the S/F ratio to an extremely high level, stable immunosuppressive Tregs dominate the microenvironment, leading to profound immune suppression and immunotherapy resistance. In contrast, a relatively low S/F ratio is accompanied by an enrichment of fragile-like Tregs and a balanced Th1-type inflammatory state, which may create favorable conditions for combined PD-1 inhibitor therapy. The two contradictory clinical outcomes may essentially represent two extreme states derived from the same underlying mechanism of S/F ratio polarization.

The clinical benefits of H. pylori eradication are markedly dependent on intervention timing:

Early eradication (precancerous lesions or early gastric cancer): A meta-analysis incorporating 11 randomized controlled trials demonstrated that eradication therapy in H. pylori-infected individuals significantly reduces the risk of gastric cancer development (53).

Late-stage eradication (metastatic gastric cancer): While eliminating the pathogen can potentiate responses to immunotherapy, this intervention entails specific risks, such as transient inflammatory resurgence within the gastric mucosa, shifts in gut microbial ecology, and the compensatory proliferation of immunosuppressive populations, notably myeloid-derived suppressor cells (MDSCs), in certain individuals (54).

Furthermore, H. pylori infection exerts distinct effects on the efficacy of immunotherapy across molecular subtypes of gastric cancer. Recent multicenter studies have shown that H. pylori positivity is a favorable prognostic factor for immunotherapy in microsatellite-stable (MSS) gastric cancer (55), a subtype that is conventionally refractory to immune checkpoint blockade. The complex regulatory network underlying its subtype-specific immunomodulation remains to be further elucidated. From a translational standpoint, the stage-stratification requirement poses a major practical hurdle. We acknowledge that until a standardized serological or histological algorithm for acute-versus-chronic discrimination is prospectively validated, the full three-variable Treg-SF Score cannot be applied as a stratified biomarker. In the interim, we propose that initial validation efforts should prioritize the simplified two-variable model (S/F ratio and CD8^+^ density, with a fixed penalty for H. pylori positivity), which remains hypothesis-generating and directly testable using existing FFPE tissue cohorts with known H. pylori status. If the simplified model shows significant prognostic signal, subsequent prospective studies can incorporate the composite staging proxies described above to refine γ and recover the full informational potential of the infection-status variable.

#### 3.3.2 Synergistic immunosuppressive network of Treg subsets, TAMs, and MDSCs

In the TME of gastric cancer, fragile-like Tregs do not function independently. Instead, they may form a synergistic immunosuppressive circuit with M2-type tumor-associated macrophages (TAMs) and myeloid-derived suppressor cells (MDSCs) (26). M2 TAMs can directly inhibit CD8^+^ T cells by secreting IL-10 and TGF-β. Meanwhile, the CCL2/CCR2 axis mediated by M2 TAMs recruits Tregs and drives their polarization into fragile-like Tregs, thereby jointly establishing an immunosuppressive microenvironment. Accumulating evidence has demonstrated that M2 TAM infiltration is closely linked to resistance against PD-1 inhibitors (56, 57). Furthermore, these three immunosuppressive cell subsets show positive co-enrichment, while their prognostic values are not fully overlapping. The S/F ratio can further stratify clinical outcomes among patients with high M2 TAM infiltration, which indicates its incremental predictive value independent of TAMs (58, 59). The core advantage of focusing on Treg subsets lies in their distinct kinetic characteristics: TAM reprogramming generally takes 1 to 2 weeks to exert its effects, whereas the phenotypic shift of Tregs, reflected by the S/F ratio, can be detected as early as 1 to 2 days after treatment initiation. Hence, the S/F ratio is more suitable to serve as an early predictive biomarker for immunotherapy response (56, 60).

### 3.4 Microbiome modulation beyond H. pylori: implications for Treg plasticity

Beyond H. pylori, the gut microbiota and its metabolites play critical roles in Treg differentiation and function, offering a broader perspective for understanding the plasticity of the gastric cancer tumor microenvironment. Numerous studies have demonstrated that gut microbial metabolites can directly and indirectly regulate Treg differentiation and homeostasis via multiple signaling pathways. Short-chain fatty acids (SCFAs) are the most well-characterized microbial metabolites. Butyrate, a major product of butyrate-producing bacteria such as Clostridium spp., promotes the differentiation of peripheral Tregs (pTregs) and upregulates Foxp3 expression by inhibiting histone deacetylases (HDACs) or activating G-protein coupled receptors, including GPR109A (61, 62). Additionally, butyrate can restore impaired Treg function through the mTOR-mediated autophagy pathway in patients with myasthenia gravis, indicating its systemic modulatory effects on Treg plasticity (63).

Tryptophan metabolites represent another important class of immune regulators. The gut microbiota metabolizes tryptophan into indole-3-lactic acid (ILA), indole-3-propionic acid (IPA), and indole-3-aldehyde (IAID). These metabolites facilitate the differentiation of Tregs, IL-10^+^ Bregs, and IL-22-producing group 3 innate lymphoid cells (ILC3s), thereby maintaining intestinal and systemic immune homeostasis (64). These metabolites have also been proven to be closely associated with the development of colorectal and other cancers. Nevertheless, their roles in modulating Treg plasticity in gastric cancer remain to be further explored (65, 66).

Bile acid metabolites, including deoxycholic acid (DCA) and lithocholic acid (LCA), are generated by the gut microbiota via biotransformation of primary bile acids and exert potent immunomodulatory functions. DCA and LCA promote the differentiation of Foxp3^+^ Tregs and suppress Th1 cells. In experimental autoimmune encephalomyelitis (EAE) models, supplementation with DCA and LCA alleviates autoimmune responses in the central nervous system. In colorectal cancer associated with inflammatory bowel disease, modulation of bile acid metabolism can inhibit tumor progression by restoring the Th17/Treg balance (67).

Linoleic acid and other lipid metabolites are also involved in Treg regulation. Porphyromonas gingivalis disrupts gut microbiota homeostasis and impairs linoleic acid metabolism, thereby exacerbating colitis by inducing a Th17/Treg imbalance (68). Moreover, 9,10-dihydroxy-12Z-octadecenoic acid (9,10-DiHOME), derived from the gut microbiota, can induce Treg activity *in vitro*, and its abundance is markedly decreased in patients with colitis (69).

The potential interference of antibiotics on Treg homeostasis is particularly noteworthy in the field of cancer immunotherapy. Preclinical studies have shown that broad-spectrum antibiotics can abrogate the drug-induced modulation of Tregs (70). More importantly, antibiotic-induced gut dysbiosis has been verified to impair immunotherapy responses in colorectal cancer models, partially due to the disrupted balance between Tregs and effector T cells, as well as the reduced production of microbial metabolites such as butyrate (71, 72). Accumulating clinical evidence has confirmed that antibiotic administration correlates with reduced efficacy of PD-1/PD-L1 inhibitors. Accordingly, prior antibiotic exposure in gastric cancer patients may interfere with the production of gut microbial metabolites and further affect the accuracy of the Treg-SF score.

Currently, the Treg-SF score only incorporates three weighted variables: intratumoral Treg functional status, H. pylori infection status, and CD8^+^ T cell density. Gut microbial metabolites and antibiotic exposure have not been integrated into the present model. In future research, specific microbial metabolites detected in serum or feces (e.g., butyrate, DCA, and indole derivatives) can be introduced as additional covariates to improve the predictive accuracy for immunotherapy outcomes. Meanwhile, evaluating the confounding effects of antibiotic history on Treg plasticity helps distinguish the influences of endogenous microbial regulation and iatrogenic interventions. These extended research directions will establish a more comprehensive biological framework for precision immunotherapy of gastric cancer.

## 4 Proposed conceptual biomarker: Treg stability-fragility score

The functional heterogeneity of Tregs indicates that total Treg abundance alone is not a reliable biomarker for immunotherapy response. Accordingly, this study proposes the Treg Stability-Fragility (Treg-SF) Score, a multidimensional biomarker integrating Treg functional status, H. pylori infection status, and effector T-cell density.

### 4.1 Definition and statistical rationale of the scoring system

The Treg-SF Score can be established within a weighted Cox proportional hazards regression framework, with variable weights calibrated via LASSO-Cox regression based on prospective cohort data. The conceptual formula is defined as:

Treg-SF Score = (S/F ratio × α) - (logCD8^+^ Tcell density × β) + (H. pylori status × γ)

α (Weight of S/F ratio): 0.6–1.2, referenced to the hazard ratios of Treg subsets for gastric cancer prognosis (58, 73, 74);

β (Weight of CD8^+^ T cell density): 0.4–0.8, referenced to the effect sizes of gastric cancer immune scores reported in published literature (73);

A critical challenge in assigning γ is that no validated biomarker currently exists to reliably distinguish acute from chronic H. pylori infection in routine clinical practice; most gastric cancer patients present with chronic infections of unknown duration. To make this framework testable, we propose two complementary strategies for handling γ in the current model:

(i) Transitional clinical proxies for infection stage. In prospective validation cohorts, we recommend using a composite of (a) serum anti-CagA IgG avidity (low avidity suggests recent infection, high avidity indicates chronic colonization), (b) gastric mucosal histology scored according to the updated Sydney System (which quantifies chronic versus active inflammation), and (c) longitudinal changes in urea breath test values over serial measurements. These proxies are not definitive but can serve as surrogates for stage assignment during model calibration, with their relative weights optimized via LASSO-Cox regression in the training cohort.

(ii) Default chronic-infection assumption for clinical deployment. In the absence of these ancillary data—which will be the case for most retrospective datasets and routine diagnostics—we propose a conservative, simplified version of the model in which γ is fixed at a single negative value (e.g., −0.3 to −0.5), reflecting the epidemiological reality that nearly all gastric cancer patients with H. pylori positivity harbor chronic persistent infection. Under this simplification, the formula reduces to a two-variable score (S/F ratio and CD8^+^ density), with H. pylori status serving as a constant negative modifier rather than a patient-specific stratified variable. This simplification inevitably sacrifices biological granularity and may underestimate the pro-inflammatory effects of acute infection in the small subset of patients with recent acquisition, but it permits immediate retrospective testing of the core S/F–CD8 axis while awaiting the development of a reliable stage-discriminating companion diagnostic. The above coefficients are used for conceptual demonstration only. The definitive weights need to be calibrated via Cox regression analysis based on large-scale clinical cohorts. The present study merely proposes a theoretical model framework rather than a finalized clinical tool.

S/F ratio: The highest weight is assigned. Functional studies across multiple cancer types have confirmed that Treg functional status (e.g., the proportion of fragile Tregs) is more accurate than total Treg count in predicting immunotherapy response in cancer patients (7).

H. pylori infection status: It can be included in the scoring system as an interaction term or an independent variable. A large-sample clinical study published in the Journal of Immunotherapy of Cancer in 2022 demonstrated that H. pylori infection is an independent predictor of poor survival among patients with advanced gastric cancer treated with immune checkpoint inhibitors (ICIs) (75–77). Notably, the weight γ needs to fully distinguish the differential biological effects between acute and chronic H. pylori infections and should not be set linearly using a simple positive/negative dichotomy, but rather be finely calibrated according to the infection stage.

CD8^+^ T cell density: As a core indicator of anti-tumor immune intensity, it is included as a logarithmic term in the denominator of the formula. Pioneering studies by Galon et al. have confirmed that intratumoral CD8^+^ T cell infiltration density is one of the strongest prognostic biomarkers for solid tumors (23, 78); this conclusion has also been fully validated in gastric cancer-related meta-analyses and can serve as an independent core variable in the scoring model (79–81).

Quantitative counting method: It is recommended to stain formalin-fixed, paraffin-embedded (FFPE) tumor tissue sections using multiplex immunohistochemistry (mIHC), perform automated counting of positive cells in specific tumor regions with pathology digital analysis software, and finally calculate the result as “the number of positive cells per square millimeter”.

**Figure 3:**
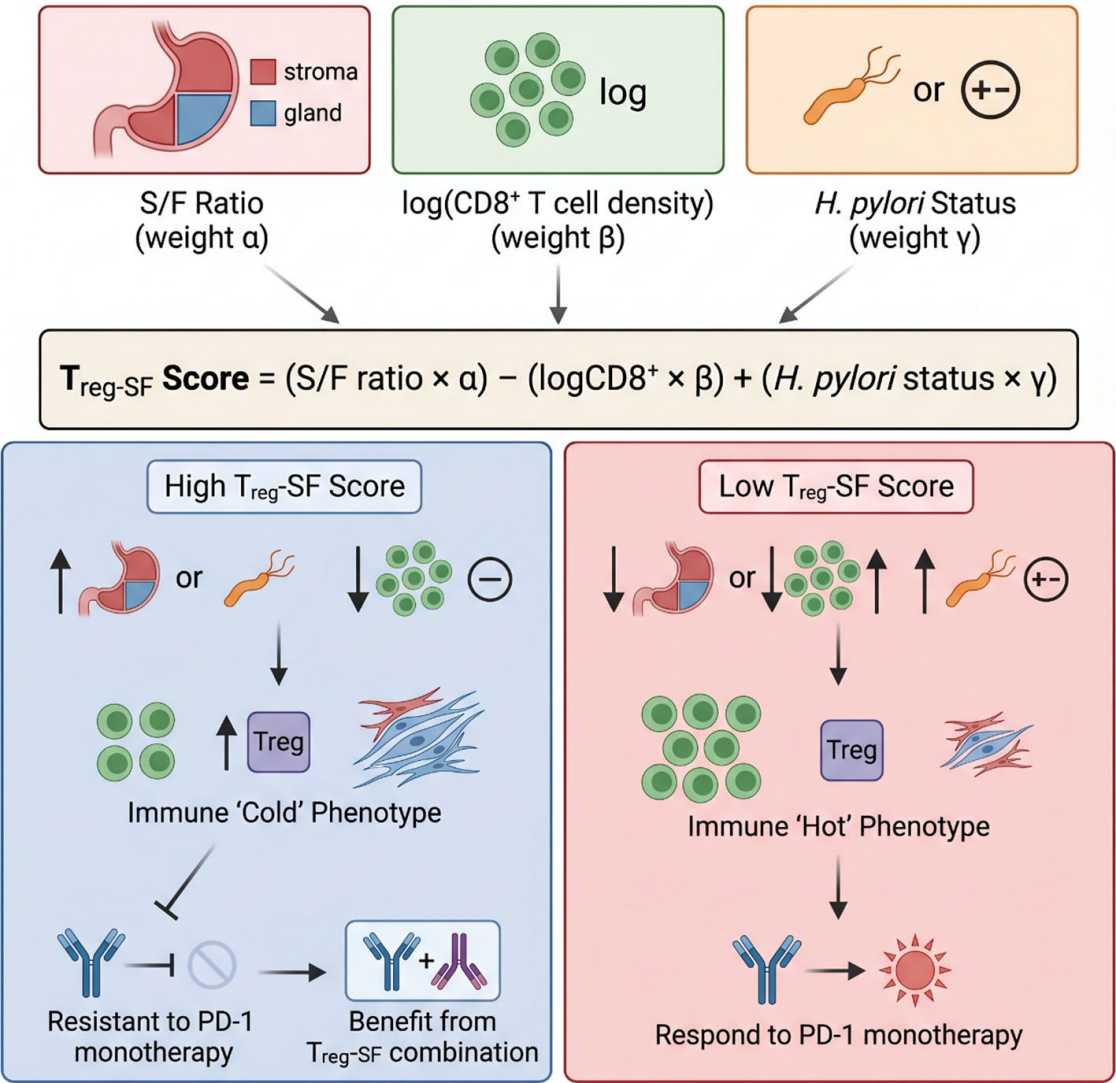
Treg Stability−Fragility (Treg−SF) score framework.

The Treg−SF Score is a novel, multidimensional conceptual biomarker designed to assess the functional competence of regulatory T cells (Tregs) rather than merely quantifying their abundance (**Figure 3**). This unified predictive framework synthesizes three distinct, independently weighted parameters: the suppressor-to-effector (S/F) ratio, the logarithmic density of intratumoral CD8^+^ T lymphocytes, and *H. pylori * infection status. Mathematically, the score is formulated as: Treg−SF Score = (S/F ratio × α) – (logCD8^+^ T cell density × β) + (H. pylori status × γ), wherein the weighting coefficients (α, β, γ) are optimized via LASSO−Cox regression analysis using prospective cohort data. The S/F ratio is assigned predominant weight, reflecting evidence that the functional state of Tregs is a superior predictor of immunotherapeutic efficacy compared to absolute Treg counts. Concurrently, the logarithmic transformation of CD8^+^T cell density—a validated proxy for antitumor immune vigor—is incorporated to accurately model immune effector potential. *H. pylori* status is integrated as an independent covariate, necessitating stratification by infection stage to delineate the divergent biological impacts of acute versus chronic colonization. Clinically, individuals exhibiting a low Treg−SF Score display an immune “hot” phenotype, characterized by a diminished S/F ratio and robust CD8^+^ infiltration, thereby predicting a favorable response to PD−1 inhibitor monotherapy. In contrast, those with an elevated Treg−SF Score exhibit an immune “cold” profile, characterized by a high S/F ratio and sparse CD8^+^ T cells, suggesting they are optimal candidates for Treg−targeted combination therapies. Designed for implementation via multiplex immunohistochemistry on standard formalin−fixed paraffin−embedded (FFPE) tissue sections, this scoring system further recommends supplementary TSDR methylation analysis to preclude the misclassification of pseudo-fragile Treg phenotypes. In addition to Treg subsets, multiple molecular signatures have been widely used to classify immune “hot” and “cold” tumors. For instance, necroptosis-associated lncRNA signatures can effectively distinguish tumor immune subtypes and predict patient prognosis. This strategy is complementary to the immune classification based on functional Treg subsets proposed in the present study, further demonstrating the universal applicability of multi-dimensional immune biomarkers across different cancer types 35965557.

### 4.2 Technical implementation and standardization

The quantitative detection of the Treg-SF score can be achieved via multiplex immunohistochemistry (mIHC) on routine formalin-fixed paraffin-embedded (FFPE) tumor tissue sections, without the need for special detection equipment, making it suitable for routine clinical application. The recommended core marker panel for accurate quantification and subtyping is as follows:

CD4 (a specific marker for helper T cells, used to delineate the T cell population)

Foxp3 (a specific marker for total Tregs, distinguishing Tregs from other immune cells)

T-bet (a characteristic marker for fragile Tregs, used to identify pro-inflammatory Treg subsets)

IFN-γ (a functional validation marker for fragile Tregs, assisting in confirming the activated state of Tregs)

CD8 (a marker for effector T cells, used to evaluate the intensity of anti-tumor immune responses)

It is important to note that studies have shown that metabolites can regulate Treg stability through epigenetic mechanisms. For example, the abnormal accumulation of 2-hydroxyglutarate can induce hypermethylation of the Foxp3 gene locus, thereby inhibiting Foxp3 transcription and expression (82, 83). Therefore, to accurately assess Treg stability (and avoid misjudgment of “pseudo-fragile” phenotypes), it is recommended to supplement the above mIHC detection with TSDR (Treg-specific demethylated region) methylation analysis based on sequencing technology to further verify the functional status and stability of Tregs, ensuring the accuracy and reliability of the score. Overall, existing studies have reported that the area under the curve (AUC) for prognostic prediction based on total TAM infiltration is approximately 0.65–0.7, while that for total Treg infiltration ranges from 0.6 to 0.65. As a marker focusing on functional Treg subsets, the S/F ratio is expected to achieve superior predictive performance compared with total Treg density. Moreover, combining the S/F ratio with TAM biomarkers can further improve predictive efficacy. These findings demonstrate the distinct incremental clinical value of targeting specific Treg subsets (84).

To ensure reproducibility, it is recommended to implement the following standardized procedures:

1. Perform standardized staining on an automated platform using validated antibody clones to ensure consistent staining intensity and uniform positive judgment criteria.
2. Two independent practicing pathologists conduct a blind evaluation of the results to perform an inter-observer consistency assessment, ensuring an overall Kappa (κ) value > 0.885 (85).
3. In the training cohort, determine the diagnostic/prognostic cut-off values for each marker and the final score by plotting ROC curves and calculating the Youden index (86).

### 4.3 Clinical application and prospective validation strategy

Patients with a low Treg-SF score (low S/F ratio and high CD8^+^ T cell density) present an immune microenvironment prone to “hot tumors” with potent endogenous antitumor immunity, and are likely to achieve favorable responses to standard PD-1 monotherapy. Patients with a high Treg-SF score (high S/F ratio and low CD8^+^ T cell density) exhibit an immunosuppressive “cold tumor” phenotype, and are expected to benefit the most from Treg-targeted combination therapy. In addition, the Treg-SF score can be integrated into knowledge graph-based clinical decision support systems in the future. Drawing on GraphRAG architectures such as RSA-KG, we can establish semantic associations among multimodal features, including the S/F ratio, CD8^+^ T cell density, and H. pylori status, as well as clinical guidelines and medication evidence, to enable automated treatment recommendations and risk alerts. Compared with standalone Cox regression models, this architecture is capable of incorporating more clinical heterogeneous factors, thereby improving the interpretability and clinical applicability of decision-making (87). Importantly, the transitional staging proxies (serology, histology, and serial breath test changes) proposed in Section 4.1 are not intended as definitive diagnostic criteria; rather, they are candidate covariates whose weights and cutoffs must be empirically calibrated in the training cohort (n = 300) and subsequently validated in the internal and external cohorts. Their predictive contribution to the overall Treg-SF Score will be assessed by comparing model fit (AIC and C-index) between the simplified fixed-γ model and the stage-stratified model incorporating these proxies.

A three-phase validation strategy is proposed in this study:

Sample size estimation follows the methodological framework for external validation published by Riley et al. in BMJ (88). This framework requires a sufficiently large sample size to precisely quantify model discrimination, calibration, and clinical utility, rather than relying on simple empirical rules such as “at least 100 events and 100 non-events”. Based on this framework:

1. Retrospective training: LASSO-Cox regression is applied to optimize variable weights in a single-center gastric cancer immunotherapy cohort (n = 300);
2. Internal validation: Prognostic and predictive performance is validated in an independent internal cohort (n = 200);
3. External multicenter validation: Validation is performed across three independent international cohorts (total n = 1000) to accurately evaluate model discrimination (89), calibration slope, and clinical utility. Model performance is further compared with established biomarkers (PD-L1 CPS, MSI-H, TMB) using the C-index and decision curve analysis.

### 4.4 Limitations

The key limitations of the Treg-SF score include:

Differences in mIHC staining and quantification exist between different platforms and laboratories, and standardized reporting in accordance with the STORMI international consensus guidelines is required to facilitate cross-study comparison and validation (90); Lack of a unified cut-off value, which needs to be calibrated via ROC curves and Youden index during the external validation phase; Difficulty in capturing the dynamic changes of Treg status during treatment—dynamic immune monitoring studies suggest that baseline static immune status is difficult to predict treatment response, and early fluctuations of peripheral blood Tregs are potential indicators for predicting the efficacy of immunotherapy in gastric cancer (91);

It may be confounded by other immune cell subsets (such as MDSCs and M2-type macrophages) (92).

## 5 Therapeutic strategies targeting Treg plasticity

Based on the bimodal model, this study proposes three complementary therapeutic strategies to shift the Treg balance toward fragility (1): upstream etiological intervention via H. pylori eradication (2); selective depletion of stable suppressive Tregs (3); pharmacological induction of Treg fragility.

**Figure 4:**
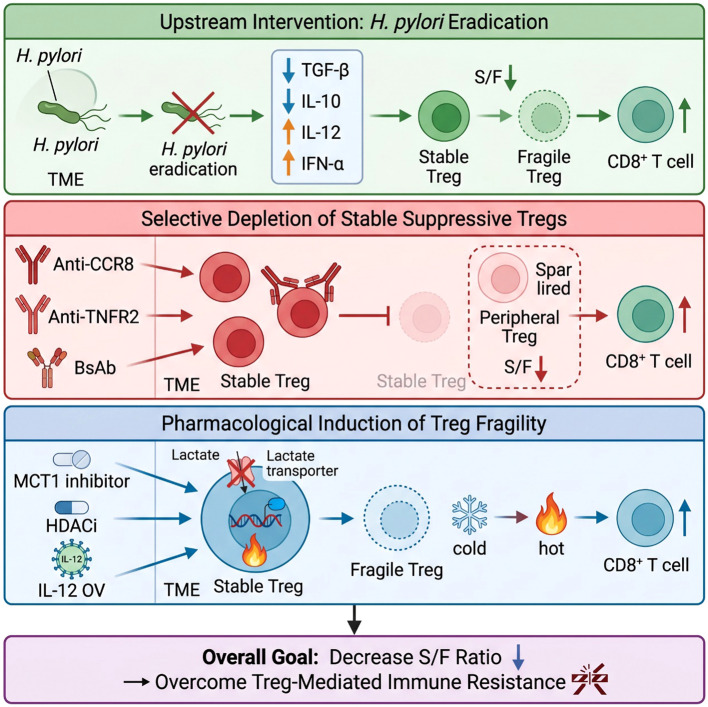
Three complementary strategies targeting Treg plasticity in gastric cancer.

Three mechanistically divergent therapeutic paradigms are advanced to destabilize the regulatory T cell (Treg) equilibrium, specifically fostering fragility to lower the stable-to-fragile (S/F) ratio (**Figure 4**). The initial approach entails upstream intervention via *H. pylori * eradication, capitalizing on the pathogen’s specific etiological contribution to gastric carcinogenesis. By eliminating the bacterium, chronic signaling through TGF−β and IL−10—critical for maintaining Treg stability—is attenuated, while the predominance of IL−12 and IFN−α is reinstated. This recalibration controllably skews the S/F ratio toward a fragile state, consequently enhancing tumor susceptibility to immunotherapeutic agents. The second paradigm focuses on the selective ablation of stable, suppressive Tregs utilizing agents directed against tumor-associated markers. Candidates include anti−CCR8 monoclonal antibodies (e.g., LM−108, CHS−114),anti−TNFR2 antibodies, and bispecific constructs (e.g., CCR8×4−1BB). Such modalities seek to purge intratumoral immunosuppressive Tregs while sparing peripheral homeostatic populations, thus mitigating the risk of systemic autoimmunity. The third strategy employs pharmacological agents to induce Treg fragility without causing global depletion. For instance, metabolic inhibitors like MCT1 blockers interrupt lactate-dependent Treg differentiation; selective epigenetic modulators, such as HDAC inhibitors, compromise Treg suppressive capacity; and IL−12-engineered oncolytic viruses provide localized pro-inflammatory cues that drive Treg conversion toward a fragile phenotype. Although each tactic targets a unique node within the Treg stability network, they share a unified goal: reversing Treg-mediated immunosuppression by diminishing the S/F ratio rather than indiscriminately eradicating the entire Treg compartment.

### 5.1 Upstream intervention: H. pylori eradication

H. pylori eradication represents a unique and underutilized strategy for modulating Treg function. Multiple real-world studies and meta-analyses have demonstrated that H. pylori-positive gastric cancer patients achieve better responses to immune checkpoint inhibitors, and H. pylori enhances immunotherapy efficacy by shaping a “hot” tumor microenvironment (92, 93). Nevertheless, the clinical benefits of H.pylori eradication for immunotherapy remain controversial across different molecular subtypes and infection stages (93–100), which warrants further prospective validation. Mechanistically, as inferred from the bimodal model, H. pylori eradication can downregulate TGF-β/IL-10 and elevate IL-12/IFN-α, thereby controllably shifting the S/F ratio toward Treg fragility and sensitizing tumors to immunotherapy.

### 5.2 Selective depletion of stable suppressive Tregs

The discovery of tumor-specific Treg markers enables therapeutic strategies that preserve peripheral homeostatic Tregs while depleting Tregs within the tumor microenvironment.

1. Anti-CCR8 monoclonal antibodies: LM-108 and CHS-114 are representative agents of this class. Phase I/II studies of LM-108 showed an overall ORR of 36.1% in gastric cancer patients receiving combination therapy with anti-PD-1 agents, while the ORR reached 87.5% in the CCR8 high-expression subgroup (1 CR and 6 PR among 8 patients) (101). CHS-114 selectively reduces intratumoral Tregs while sparing CCR8-negative Tregs and effector T cells. Treatment of tumor-bearing human CCR8 knock-in (huCCR8KI) mice with CHS-114 significantly inhibited tumor growth by 62.6% (102).
2. Anti-TNFR2 antibodies: Nanobody-161 is a novel non-blocking TNFR2 antagonist that suppresses tumor growth without causing systemic immunosuppression, representing a promising candidate for safe and effective solid tumor therapy (103).
3. Bispecific antibodies: The 4-1BB×CCR8 bispecific antibody FRP303 and TNFR2×CCR8 bispecific antibody FT10-Fab exhibit potent antitumor activity in preclinical models by eliminating tumor-infiltrating Tregs and enhancing the effector function of CD8^+^ T cells (104). The HER2×CCR4 DVD-Ig bispecific antibody enhances antitumor immune responses against HER2-positive tumors in preclinical models via chemokine blockade and ADCC-mediated clearance of tumor-associated Tregs (105).

**Figure 5:**
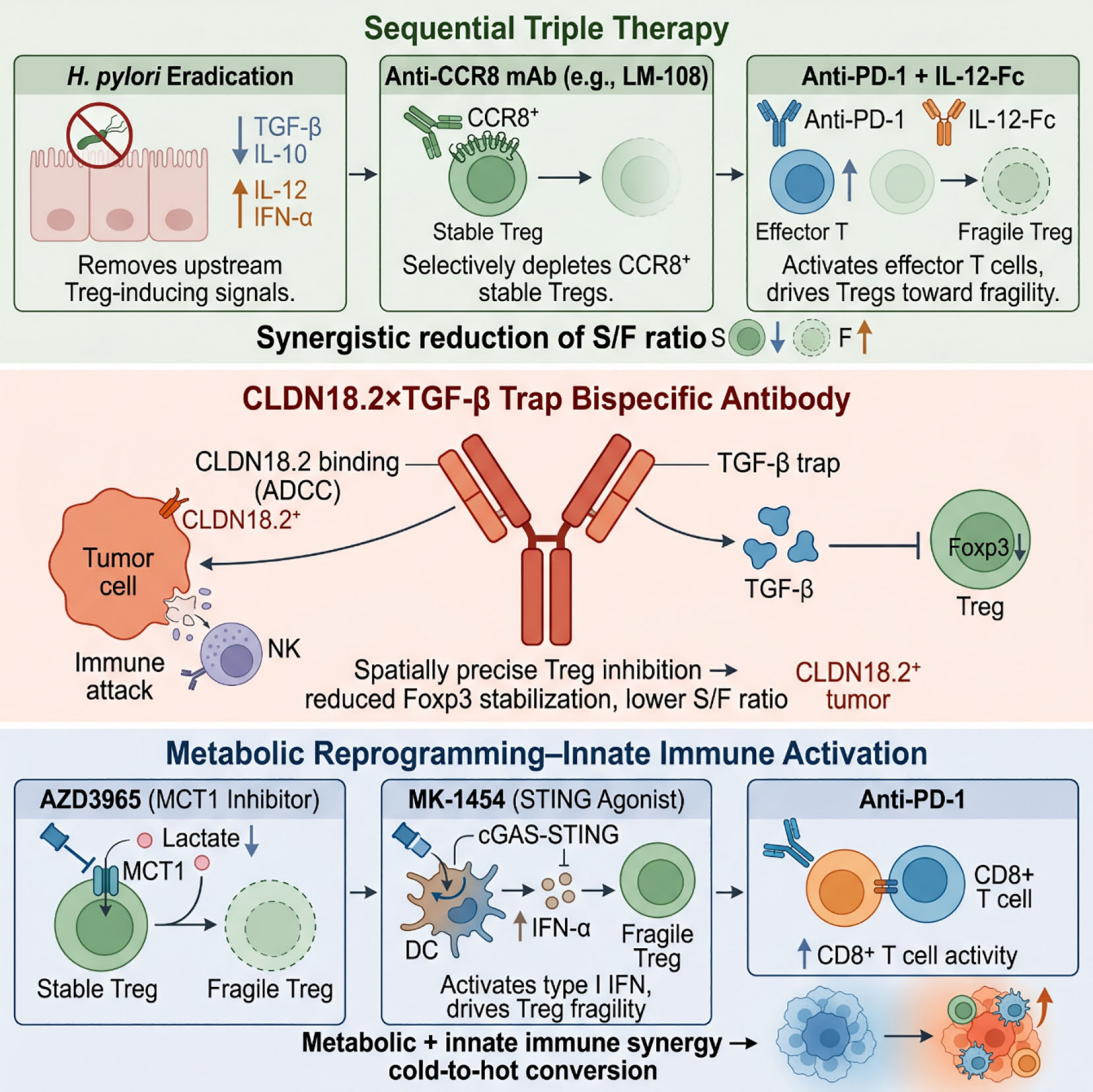
Proposed combination therapeutic regimens.

Given that monotherapies aimed at modulating Treg plasticity are often undermined by compensatory immunosuppressive pathways, we propose three rationally engineered combination strategies (**Figure 5**). The first regimen outlines a sequential triple therapy tailored for patients with unresectable advanced gastric cancer who have not yet undergone gastrectomy. This approach initiates with H. pylori eradication to eliminate upstream triggers of Treg induction, thereby shifting the baseline stability-to-fragility (S/F) ratio toward a more fragile state. Subsequent administration of an anti-CCR8 monoclonal antibody targets the depletion of the predominant CCR8^+^ stable Treg subset. The final phase combines anti-PD-1 blockade with an IL-12-Fc fusion protein, a dual strategy designed to reactivate effector T cells while concurrently pushing remaining Tregs toward functional instability. The second proposal involves a hypothetical bispecific antibody integrating CLDN18.2 targeting with a TGF-β trap, merging two established therapeutic mechanisms for spatially precise Treg modulation. The CLDN18.2-binding domain facilitates antibody-dependent cellular cytotoxicity against gastric tumor cells and ensures localized accumulation within the tumor microenvironment. Simultaneously, the TGF-β trap moiety sequesters bioactive TGF-β, thereby inhibiting Smad3/4-mediated Foxp3 stabilization and dampening Treg-driven immunosuppression. The third regimen represents a sequential protocol that couples metabolic reprogramming with innate immune activation, using the MCT1 inhibitor AZD3965, the STING agonist MK-1454, and a PD-1 checkpoint inhibitor.AZD3965 interferes with lactate metabolism specifically within Tregs, whereas MK-1454 stimulates the cGAS-STING axis in dendritic cells and macrophages, promoting type I interferon secretion and inducing Treg fragility. Concurrent PD-1 inhibition further unleashes the antitumor potential of CD8^+^ effector T cells. Collectively, these three strategies aim to synergistically lower the S/F ratio; however, each demands rigorous preclinical scrutiny followed by prospective clinical assessment to validate efficacy and safety.

### 5.3 Pharmacological induction of Treg fragility

The strategy proposed in this section does not aim to eliminate all Tregs. Instead, it synergistically weakens the competitive advantage of immunosuppressive Tregs from metabolic, epigenetic, and inflammatory signaling dimensions, drives their conversion toward a fragile phenotype, reduces the S/F ratio, and promotes antitumor immune responses.

1. Metabolic inhibitors: MCT1 inhibitors block lactate uptake and suppress lactate-dependent Treg differentiation and expansion in the tumor microenvironment (106). Fatty acid oxidation inhibitors inhibit Treg differentiation *in vitro*, whereas genetic models confirm that such effects are independent of the Cpt1a target and may involve alternative mechanisms such as mitochondrial respiration (107).

2. Epigenetic modulators: Epigenetic modifications exert bidirectional regulation on Treg stability. Pan-HDAC inhibitors enhance Foxp3 stability and Treg suppressive function, while selective isoform inhibitors (such as HDAC6 inhibitors and Class I HDAC inhibitors) can selectively impair Treg function (108, 109). BRD4 plays an essential role in Treg development; the capacity of BRD4 inhibitors to drive Treg toward a pro-inflammatory phenotype remains to be further validated (110).

3.IL-12-armed oncolytic viruses: These viruses selectively replicate within the tumor microenvironment and locally secrete IL-12.They reduce Treg abundance and suppressive activity, drive the conversion of immunosuppressive Tregs into fragile-like Tregs, and increase the infiltration of activated CD8^+^ T cells, thereby converting “cold” tumors into “hot” ones (111).

## 6 Proposed combination regimens: rationale, risks, and preclinical validation

Relying solely on therapies directed at regulatory T cells (Tregs) often results in suboptimal clinical outcomes, as the tumor microenvironment frequently restores immunosuppressive conditions via compensatory pathways, thereby precipitating therapeutic failure or the swift emergence of acquired drug resistance. To address these challenges, this investigation introduces three synergistic combination strategies grounded in distinct mechanistic principles, accompanied by comprehensive protocols for preclinical validation and a rigorous evaluation of potential risks.

### 6.1 Regimen 1: sequential triple therapy

(H. pylori eradication → anti-CCR8 → anti-PD-1 + IL-12-Fc)

Target population: This indication is strictly confined to individuals presenting with unresectable, advanced, or metastatic gastric carcinoma who have not yet undergone surgical resection; consequently, it is not indicated for adjuvant therapy following total gastrectomy. Rationale: The sequential administration strategy aims to maximize efficacy while minimizing toxicity:

1. H. pylori eradication: Retrospective clinical studies indicate that H. pylori positivity is an independent adverse prognostic factor for immunotherapy in advanced gastric cancer (OS HR = 2.85) (76). Eradication removes the upstream Treg-inducing signal, remodels the immune microenvironment, and shifts the baseline S/F ratio toward Treg fragility.
2.Anti-CCR8 therapy: A phase I/II trial in PD-1-resistant gastric cancer demonstrated that LM-108 combined with anti-PD-1 antibody achieved an ORR of 87.5% and a DCR of 100% in the CCR8-high subgroup with an acceptable safety profile (101). These findings support anti-CCR8 as a core step for depleting dominant, stable Treg populations.
3.Anti-PD-1 + IL-12-Fc: Preclinical models have validated that engineered attenuated IL-12-Fc fusion protein combined with PD-1 blockade exhibits potent antitumor activity and an improved therapeutic index (112). This combination activates effector T cells and drives residual Tregs toward a fragile phenotype.

Potential risks: Systemic IL-12-related toxicity, gastrointestinal toxicity, and immune-related adverse events.

### 6.2 Regimen 2: CLDN18.2×TGF-β trap bispecific antibody (hypothetical)

Rationale: This hypothetical bifunctional molecule integrates two validated therapeutic modalities to achieve spatially targeted Treg regulation (113, 114).

CLDN18.2 arm: Mediates ADCC-dependent killing of gastric cancer cells and enables specific targeting to the tumor microenvironment.

TGF-β trap arm: Sequesters local TGF-β, theoretically blocks Smad3/4 signaling to prevent Foxp3 stabilization, and attenuates Treg-mediated immunosuppression.

Potential risks: Off-tumor toxicity, immunogenicity, manufacturing difficulty, and lack of efficacy in CLDN18.2-negative tumors.

### 6.3 Regimen 3: metabolic reprogramming-innate immune activation sequential therapy

(AZD3965 + PD-1 inhibitor + MK-1454)

Rationale: The triple combination targets three complementary pathways:

1. AZD3965 (MCT1 inhibitor): Disrupts lactate metabolism in Tregs (115). Its Phase I trial in solid tumors (NCT01791595) has been completed (116), providing direct clinical dosing experience. Notably, this agent also interferes with CD8^+^ T cell metabolism. The net therapeutic outcome (overcoming Treg dominance versus impairing effector T cells) requires rigorous evaluation in further *in vitro* and *in vivo* studies.
2. MK-1454 (STING agonist): Activates dendritic cells and macrophages to induce robust interferon production via the cGAS-STING pathway, which theoretically drives Treg fragility (117–119).
3. PD-1 inhibitor: Releases the antitumor activity of effector T cells with a well-established theoretical basis and solid clinical evidence in gastric cancer.

Potential risks: Lactic acidosis and immune-related adverse events.

We propose the following criteria to judge whether the therapeutic benefits of MCT1 inhibition against Tregs outweigh its potential impairment of effector T cell function: firstly, the tumor lactic acid concentration threshold. When the lactic acid concentration in the tumor microenvironment exceeds 10 mM (indicating a highly glycolytic phenotype), MCT1 inhibition primarily eliminates lactate-dependent Tregs (120). CD8^+^ T cells can compensate for energy requirements via glutamine metabolism and other pathways, resulting in a net positive immunological effect. By contrast, this therapy is not recommended for tumors with low lactic acid levels, as CD8^+^ T cells will suffer more severe metabolic damage (120, 121). Secondly, MCT1 expression ratio between cell subsets. Single-cell transcriptomics can be used to quantify the expression ratio of SLC16A1 (MCT1) between tumor-infiltrating Tregs and CD8^+^ T cells. A ratio greater than 2 indicates that MCT1 inhibition exerts a markedly stronger killing effect on Tregs than on effector T cells, resulting in a superior benefit-risk profile (120, 122). Finally, optimization of the administration schedule.

A sequential treatment regimen is recommended: MCT1 inhibitors are administered for two days to deplete Tregs, followed by the combination of STING agonists and PD-1 inhibitors. This strategy takes advantage of the metabolic fragility of Tregs and reserves time for metabolic adaptation of CD8^+^ T cells, thereby reducing damage to effector cells (123, 124).

### Reference to spatial metabolomics approaches

Integrated analytical pipelines combining single-cell and spatial transcriptomics can be adopted to quantify cellular metabolic activity across distinct tumor regions and precisely predict the regional effects of MCT1 inhibition (24, 125, 126). For instance, regions at the invasive margin enriched with Tregs gain more benefits, while the tumor core with abundant CD8^+^ T cells faces a higher risk of cellular injury. This analytical strategy has been widely applied in studies on metabolic modulation for immunotherapy.

For mechanistic deconvolution of multi-agent combination regimens, AI-enabled network pharmacology is a viable approach. By constructing heterogeneous "drug-target-immune cell" networks, we can systematically quantify the contribution of each component to core endpoints such as the S/F ratio and CD8^+^ T cell activation. This method greatly reduces the workload of preclinical validation, helps prioritize core therapeutic components, and optimizes the dosage proportion of combined regimens (127).

## 7 Limitations

1. Absence of prospective validation for central hypotheses

The bimodal framework, S/F ratio, and Treg SF score function as conceptual tools designed to generate hypotheses. All corroborating data stem from retrospective re-examination of existing single-cell RNA sequencing datasets; these metrics have not yet been formally quantified or established as prognostic or predictive biomarkers in any forward-looking gastric cancer cohort. Furthermore, the three combination therapeutic strategies outlined here remain hypothetical constructs, necessitating rigorous assessment through clinical trials to determine their translational potential.

2. Existing approaches, such as multiplex immunohistochemistry (mIHC) and single-cell RNA sequencing (scRNA-seq), merely delineate the regulatory T cell (Treg) landscape at isolated temporal intervals. Consequently, the dynamic trajectory of the S/F ratio throughout the progression of *H. pylori* infection and subsequent therapeutic modulation remains elusive, necessitating validation through longitudinal *in vivo* imaging coupled with single-cell lineage-tracing methodologies.

3. The composition and functional status of regulatory T cells (Tregs) are significantly influenced by patient-specific heterogeneity—including age, sex, and tumor stage—as well as temporal variations, therapeutic histories, and concurrent comorbidities. Current retrospective analyses have failed to sufficiently adjust for these covariates, potentially obscuring the genuine correlation between the stable-to-fragile (S/F) Treg ratio and clinical prognosis.

4. Functional attributes of Treg subsets exhibit distinct spatial heterogeneity across tumor microcompartments, such as the invasive margin, tumor core, and tertiary lymphoid structures (TLS). While this review hypothesizes that fragile Tregs preferentially inhabit TLS with specific functional implications, this premise necessitates rigorous validation through spatial transcriptomics and multiplexed imaging to map their distribution and activity across discrete microanatomical niches.

5. Confounding effects of other immunosuppressive cells: Tumor-associated macrophages (TAMs) and myeloid-derived suppressor cells (MDSCs) also exert prominent immunosuppressive roles in the gastric cancer microenvironment. Their interplay with Tregs—whether synergistic, competitive, or independent—remains unclear. These cellular subsets may influence immunotherapy response independently of the S/F ratio, acting as potential confounders for the Treg-SF scoring system. When optimizing the scoring model in the future, the proportion of M2-type TAMs and MDSC density should be incorporated as covariates to further improve predictive accuracy. Meanwhile, therapeutic strategies combining Treg targeting with TAM reprogramming may reshape the gastric cancer tumor microenvironment more effectively than single-target interventions.

6. Insufficient functional validation of gastric cancer-specific fragile Tregs: Mechanistic insights into fragile Tregs are predominantly derived from melanoma and breast cancer models. Although scRNA-seq data have confirmed the presence of Foxp3low T-bet+ Tregs in gastric cancer, their suppressive capacity, cytokine secretion dynamics, and lineage stability within the gastric tumor microenvironment lack direct functional evidence. *In vitro* co-culture suppression assays and *in vivo* lineage tracing studies are required to confirm their proinflammatory properties and rule out the possibility that they merely represent a transient intermediate state during Treg conversion toward Th1-like effector cells.

7. Unresolved stratification of *H. pylori* infection stage. The bimodal model posits opposite biological effects for acute versus chronic *H. pylori* infection, yet no consensus biomarker or clinically available assay can reliably assign individual patients to either stage. Until such a companion diagnostic (e.g., avidity-based serology, multiplex cytokine profiling, or methylation-based bacterial strain typing) is developed and externally validated, the γ coefficient in the Treg-SF formula cannot be individually calibrated. The simplified fixed-γ version of the score proposed in Section 4.1 represents a pragmatic workaround, but it inherently reduces model specificity and may obscure the potential immunostimulatory effects of acute infection in a minority of patients. This limitation must be clearly communicated in any future clinical application of the score.

8. Three key uncertainties must be confronted before the bimodal model can be translated. First, no consensus biomarker currently discriminates acute from chronic *H. pylori* infection, rendering the S/F ratio uninterpretable in individual patients. Second, MCT1 inhibition to destabilize Tregs is inherently self-contradictory: lactate also fuels effector CD8^+^ T cells, and whether the net immunological effect is beneficial remains unknown. Third,fragile Tregs in gastric cancer lack direct functional validation; the Foxp3low T−bet+ subset could simply be transitional Th1-like intermediates, a possibility resolvable only by lineage tracing. These gaps do not invalidate the hypothesis but define the prospective validation agenda. Another major limitation of the current model is that the immunosuppressive function of fragile-like Tregs has not been validated through *in vivo* functional assays with gene-editing technology in gastric cancer. Existing inferences are mainly based on the phenotypic conservation across different cancer types. The above analytical approaches, including RNA velocity analysis, TCR clonotype tracing, and spatial colocalization analysis, can be implemented using published public datasets, which serve as feasible strategies for partial model validation without additional experiments. In future studies, CRISPR-engineered organoid co-culture systems should be adopted to further clarify the lineage stability and functional specificity of fragile-like Tregs.

## 8 Conclusion and perspective

A growing body of literature identifies gastric cancer as a paradigmatic model of Treg-mediated immune evasion. Crucially,the equilibrium between immunosuppressive stable Tregs and proinflammatory fragile subsets,rather than the sheer abundance of total Tregs, appears to dictate the host immune landscape and therapeutic outcomes. As a pivotal regulator of this dynamic, *H. pylori* infection exhibits stage-specific duality: acute infection triggers proinflammatory signaling that induces Treg fragility, whereas chronic persistence fosters Treg stabilization. This dichotomy delineates a distinct therapeutic window for upstream etiological targeting.

The unified Treg bimodal model proposed in this review establishes a testable mechanistic framework for understanding Treg heterogeneity and plasticity in gastric cancer. Both the Treg-SF score and the three combination regimens derived from this model are hypothesis-generating concepts. Integrating Treg functional status, *H. pylori* infection status, and CD8^+^ T cell density, the Treg-SF score is expected to predict immunotherapy response more accurately than existing biomarkers, including PD-L1 CPS, MSI-H, and TMB. The three combinatorial strategies target Treg plasticity from three complementary dimensions: upstream etiological intervention, selective depletion of stable Tregs, and pharmacological induction of Treg fragility. Their shared goal is to reverse tumor immunosuppression by reducing the S/F ratio, instead of non-specifically eliminating all Treg populations. All these hypotheses require rigorous preclinical validation and prospective clinical trials. Priority directions for future research include: (1) prospective clinical validation of the Treg-SF score; (2) functional characterization of gastric cancer-specific fragile-like Tregs; (3) development of tumor-specific Treg modulators.

Finally, as a highly plastic immunomodulatory subset, Tregs possess therapeutic potential far beyond tumor immunology. The bimodal model and fragility-inducing strategy proposed herein can be extrapolated in the opposite direction to autoimmune diseases, namely, restoring immune tolerance by stabilizing Treg phenotypes and inhibiting fragility. Research between the two fields can mutually inspire each other: inducing Treg fragility to break immune tolerance in tumor immunity, versus maintaining Treg stability to restore tolerance in autoimmunity. Their molecular mechanisms mirror each other, and therapeutic strategies can be mutually referenced. Systematically investigating Treg plasticity under a shared framework covering oncology and autoimmunity may broaden the landscape of precision immunotherapy.
